# The effect of Ding’s screws and tension band wiring for treatment of olecranon fractures: a biomechanical study

**DOI:** 10.1038/s41598-024-60264-7

**Published:** 2024-05-01

**Authors:** Yong Zhao, Hongbo Tian, Nuo Yin, Li Du, Mingmang Pan, Liang Ding

**Affiliations:** Department of Orthopaedics, Shanghai Jiao Tong University Affiliated Sixth People’s Hospital South Campus, Shanghai, 201400 China

**Keywords:** Olecranon fractures, Tension band wiring, Ding’s screw and tension band wiring, Biomechanical study, Complications, Bone, Trauma

## Abstract

Although tension band wiring (TBW) is popular and recommended by the AO group, the high rate of complications such as skin irritation and migration of the K-wires cannot be ignored. Ding’s screw tension band wiring (DSTBW) is a new TBW technique that has shown positive results in the treatment of other fracture types. The objective of this study was to evaluate the stability of DSTBW in the treatment of olecranon fractures by biomechanical testing. We conducted a Synbone biomechanical model by using three fixation methods: DSTBW, intramedullary screw and tension band wiring (IM-TBW), and K-wire TBW, were simulated to fix the olecranon fractures. We compared the mechanical stability of DSTBW, IM-TBW, and TBW in the Mayo Type IIA olecranon fracture Synbone model using a single cycle loading to failure protocol or pullout force. During biomechanical testing, the average fracture gap measurements were recorded at varying flexion angles in three different groups: TBW, IM-TBW, and DSTBW. The TBW group exhibited measurements of 0.982 mm, 0.380 mm, 0.613 mm, and 1.285 mm at flexion angles of 0°, 30°, 60°, and 90° respectively. The IM-TBW group displayed average fracture gap measurements of 0.953 mm, 0.366 mm, 0.588 mm, and 1.240 mm at each of the corresponding flexion angles. The DSTBW group showed average fracture gap measurements of 0.933 mm, 0.358 mm, 0.543 mm, and 1.106 mm at the same flexion angles. No specimen failed in each group during the cyclic loading phase. Compared with the IM-TBW and TBW groups, the DSTBW group showed significant differences in 60° and 90° flexion angles. The mean maximum failure load was 1229.1 ± 110.0 N in the DSTBW group, 990.3 ± 40.7 N in the IM-TBW group, and 833.1 ± 68.7 N in the TBW group. There was significant difference between each groups (*p* < 0.001).The average maximum pullout strength for TBW was measured at 57.6 ± 5.1 N, 480.3 ± 39.5 N for IM-TBW, and 1324.0 ± 43.8 N for DSTBW. The difference between maximum pullout strength of both methods was significant to *p* < 0.0001. DSTBW fixation provides more stability than IM-TBW and TBW fixation models for olecranon fractures.

## Introduction

Olecranon fractures are common fractures of the upper limb^1^. The Mayo Type IIA type is the most common type, accounting for approximately 60% of olecranon fractures^2^. The most popular operative intervention of this fracture type is tension band wiring (TBW) with Kirschner wires (K-wires)^3^, which is recommended by the AO^4^. Since TBW was first described by Weber and Vasey^5^ in 1963, its excellent outcomes have been demonstrated in multiple biomechanical and clinical studies^6–10^. However, the complications of TBW, including loss of reduction, K-wire migration and skin irritation, cannot be ignored^11–13^. In some cases, the incidence is up to 80%^11–13^.

Previous research has indicated that cannulated screws, specifically intramedullary screw (IM) and IM-TBW^14^, have been utilized in the treatment of olecranon fractures. However, studies have demonstrated that fixation with IM screws presents technical challenges and unreliability^15–17^. In contrast, the IM-TBW technique offers improved structural stability compared to fixation with IM screws alone^18,19^. Nonetheless, the IM-TBW technique necessitates the incorporation of a large washer in conjunction with steel wire to create tension bands, resulting in a higher complication rate compared to the use of screws alone^20^. Furthermore, the issue of withdrawal fixation remains unresolved in the context of IM-TBW, with reported withdrawal rates ranging from 6.6 to 11.1%^21,22^.

**Figure 1:**
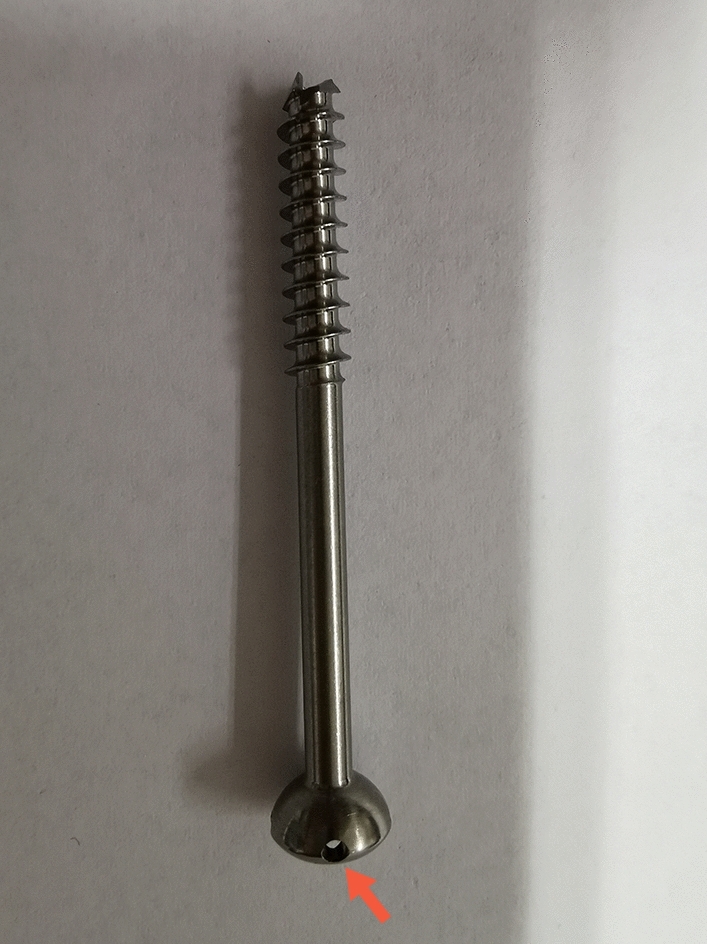
The picture of Ding’s screw. Red arrow shows the holes on the tail of Ding’s screw, allowing the 18-gauge metal wire to pass through.

Ding’s screw and tension band wiring (DSTBW), a novel TBW technique, has demonstrated promising outcomes in treating inferior pole patellar fractures^23^. The application of DSTBW technology (see Fig. 1) in olecranon fractures may mitigate the complications associated with traditional TBW. This study aims to assess the efficacy of Ding's screw and tension band wiring (DSTBW) in treating olecranon fractures through biomechanical testing.

## Materials and methods

### Biomechanical testing

**Figure 2:**
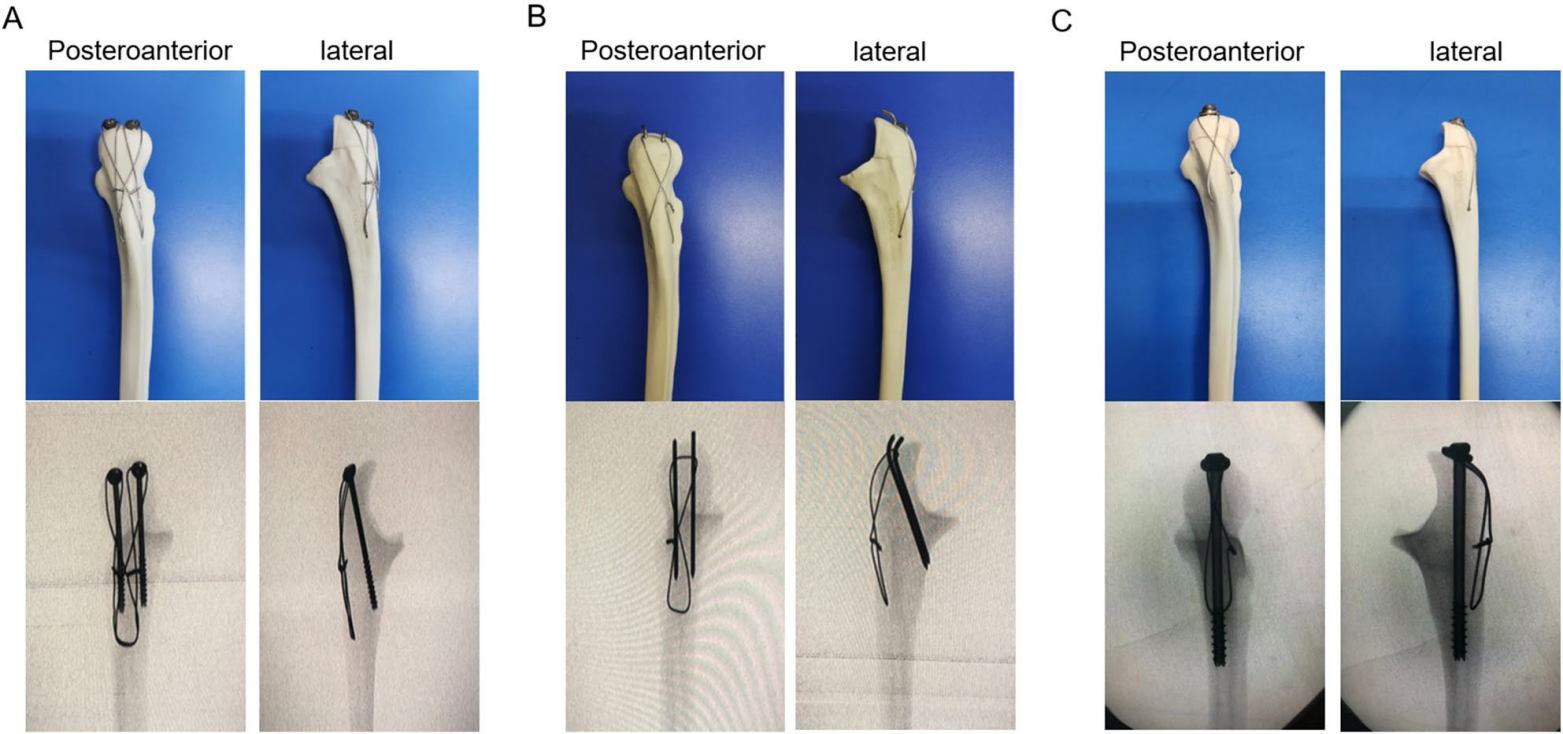
A Synbone Mayo type IIA fractures model demonstrates the fixation technique in DSTBW (**A**), TBW (**B**) and IM-TBW (**C**). (**A**, **B**, **C**) Posteroanterior and lateral views and X-ray of each group.

Forty-eight ulnar Synbone models (Synbone, Malans, Switzerland) of the same laterality, size, and density were used in this study. Mayo type IIA olecranon fracture models were created by an osteotomy. The models were then randomly divided in two groups: TBW group (n = 16), IM-TBW group (n = 16) and DSTBW group (n = 16). In the DSTBW group,the fractures were stabilized through methods reported in our previous study^16^ : the fractures were reduced and fixed by two Ding’s screws (4.0 mm in diameter); two 18-gauge metal wires were passed through the holes on the tail of Ding’s screws separately and used to make the figure-of-eight wiring between the 2.0-mm hole on the ulnar shaft and the two Ding’s screws(Fig. 2A). In the TBW group, the fractures were stabilized by the traditional K-wires and tension band fixation^24,25^ (Fig. 2B). In the IM-TBW group, the fractures were stabilized by the IM and tension band fixation^26^ (Fig. 2C).The position of internal fixation was determined by X-ray technique for each model in all groups.

**Figure 3:**
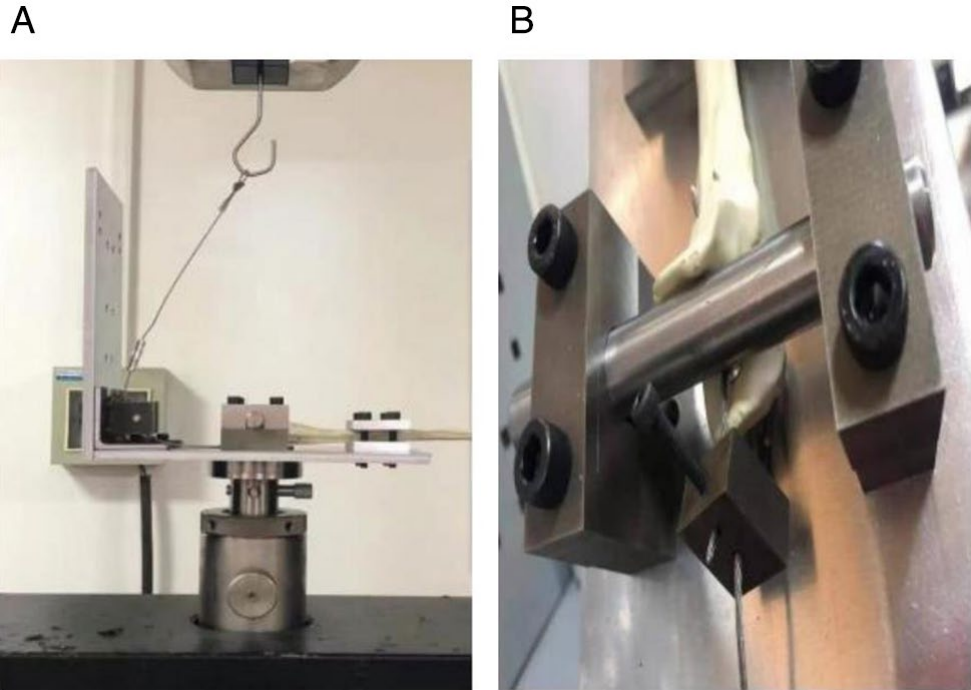
Biomechanical test set-up. (**A**)Triceps tendon forces were simulated in the material testing machine. (**B**) Shows the olecranon fracture Synbone model fixed to a jig and a metal cable attached to clamp the traction machine.

First, we randomly selected 10 models in each group of TBW, IM-TBW,and DSTBW for cyclic load test. According to a previous study^27^, all 30 models (10 TBW models,10 IM-TBW models, and 10 DSTBW models) were mounted on an axialtorsion servohydraulic fatigue testing system (Jinan Heng Rui Jin Testing Machine MRP-TM300, China) (Fig. 3). A customized fixture was used to stabilize the proximal end of the ulna on the base of the testing machine, and the triceps muscle movement when the elbow was bent 90° was simulated by pulling a 2.8 mm wire. All specimens were tested under single cycle load failure with a displacement speed of 10 mm/min. The maximum failure load was defined as the point where the load displacement curve suddenly drops, and measure the maximum failure load in two fixed technologies. In 0°, 30°, 60° and 90°flexion, tension forces (10–400 N) at a frequency of 1 Hertz (Hz) were applied 300 cycles. The loading from 10 to 150 N in 0°, from 10 to 150 N in 30°, from 10 to 250 N in 60°and from 10 to 400 N in 90°were performed. In the end, in 90°flexion load-to-failure tests were performed. Fracture gap was measured with a handheld digital caliper by 3 independent observers and the average gap calculated. The ultimate load to failure were recorded. Secondly, the method described by Mullett JH et al.^11^ was used to test the pullout force of the other 18 models (6 TBW models,6 IM-TBW models, and 6 DSTBW models) (Jinan Heng Rui Jin Testing Machine MRP-TM300, China). Each Ding’s screw has its own cerclage wire in a figure of eight, while in the classic TBW technique there are two K-wires with one cerclage wire in a figure of eight for both of them. K-wire migration often occurs in TBW, so it is interesting to compare the pullout forces of each group. In theory, the pullout strength of Ding's screw in DSTBW group is influenced by the individual cerclage wire for each screw, so it is not easy to migrate.We randomly selected a Ding’s screw or intramedullary screw or K-wire to measure the pullout force and set it to represent the pullout force of each group.Then the maximum value for both groups was recorded.

### Statistical analysis

Data were analyzed using SPSS 19.0 software (IBM Corp, Armonk, NY). Values are expressed as the means ± SD. Differences between groups were compared by one-way ANOVA. A P value of less than 0.05 was considered statistically significant.

## Results

**Figure 4:**
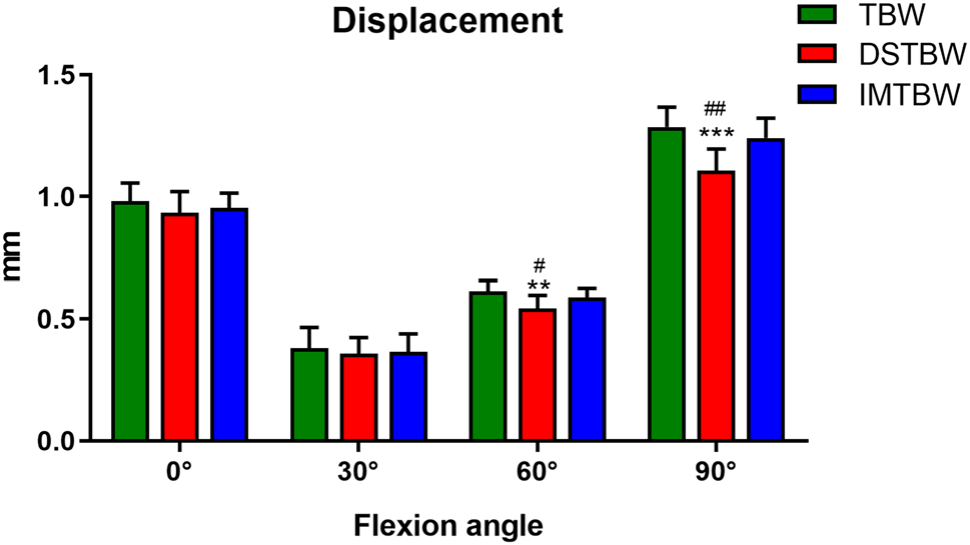
Displacement. There is less displacement in the DSTBW group than in the TBW and IM-TBW groups. (*means compared with the TBW group; ^#^ means compared with IM-TBW group; **p < 0.01, ***p < 0.001, ^#^*p* < 0.05, ^##^*p* < 0.01).

**Figure 5:**
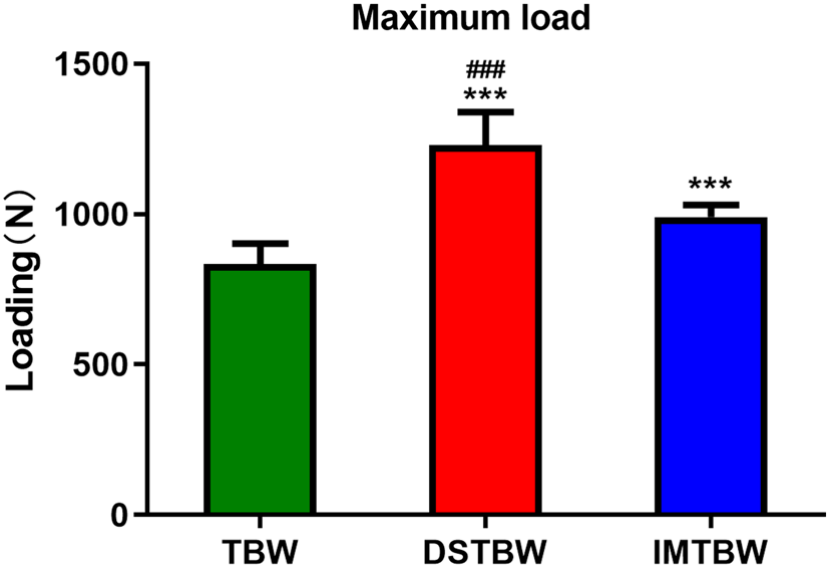
Loading for failure. (* means compared with the TBW group; # means compared with IM-TBW group; ***p < 0.001, ^###^*p* < 0.001).

**Figure 6:**
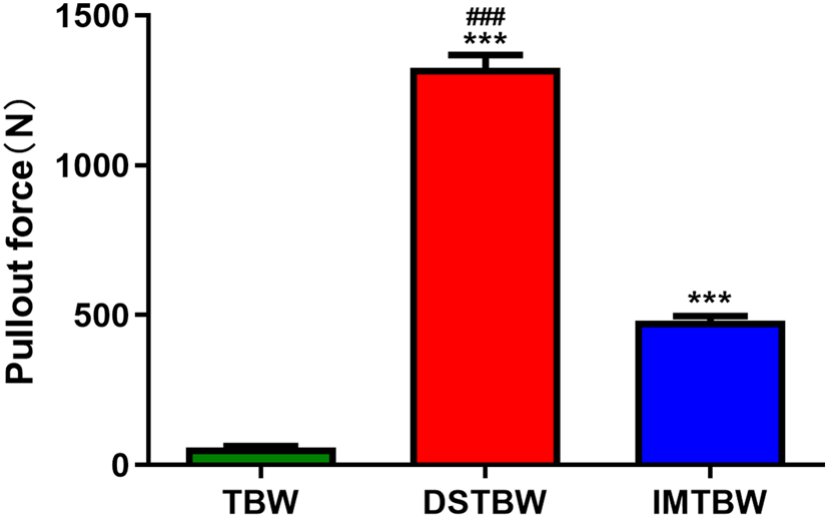
The biomechanical study of pullout force. (*means compared with the TBW group; # means compared with IM-TBW group; ***p < 0.001, ^###^*p* < 0.001).

All specimens were subjected to load-to-failure tests, with gap formation measured at 0°, 30°, 60°, and 90° flexion under 300 cycles (see Fig. 4). The average fracture gap for the TBW group was 0.982 mm, 0.380 mm, 0.613 mm, and 1.285 mm at each respective flexion angle. Similarly, the IM-TBW group exhibited average fracture gaps of 0.953 mm, 0.366 mm, 0.588 mm, and 1.240 mm, while the DSTBW group showed average fracture gaps of 0.933 mm, 0.358 mm, 0.543 mm, and 1.106 mm at the same flexion angles.During the cyclic loading phase, no specimens in any group experienced failure. The displacement of the DSTBW group was lower than that of the TBW and IM-TBW groups, statistical analysis revealed the differences in flexion grades between the groups (DSTBW VS TBW 0°: *p* = 0.154, 30°: *p* = 0.521, 60°: *p* = 0.002, 90°: *p* = 0.000; DSTBW VS IM-TBW 0°: *p* = 0.555, 30°: *p* = 0.815, 60°: *p* = 0.037, 90°: *p* = 0.001). Compared with the IM-TBW and TBW groups, the DSTBW group showed significant differences in 60° and 90° flexion angles. The mean maximum failure load was 1229.1 ± 110.0 N in the DSTBW group, 990.3 ± 40.7N in the IM-TBW group, and 833.1 ± 68.7 N in the TBW group (Fig. 5). A significant difference was observed between the groups (*p* < 0.001).The failures of fixed structures were observed exclusively at the osteotomy site. The failure of TBW can be attributed to the migration of the K-wire, whereas the failure of DSTBW fixation is a result of the crushing of synbone caused by the steel wire. The average maximum pullout strength for TBW was measured at 57.6 ± 5.1N, 480.3 ± 39.5N for IM-TBW, and 1324.0 ± 43.8N for DSTBW. The difference in maximum pullout strength between the three methods was found to be statistically significant at *p* < 0.0001 (Fig. 6).

The failure of TBW can be attributed to the migration of the K-wire, whereas the failure of DSTBW and IM-TBW fixation is a result of the crushing of synbone caused by the steel wire.

## Discussion

A prevalent belief in the literature and surgeons is that TBW is an easy, effective and reliable technique, which can be performed by residents early in their training. In contrast, the followed complication rate is up to 80%^11–13^. In a retrospective study, Schneider et al.^28^ reviewed 233 patients undergoing TBW based on 2252 radiographs at five different institutions. They found that 91.21% of patients had an insufficient scuttling of K-wires, which could lead to pain complications and increase the risk of secondary dislocation^28^. In their study, the main postoperative patient complaints were the prominence of the K-wires under the skin, secondary dislocation and the proximal migration of the K-wires causing pain, perforation of the skin and local inflammation^28^. Similarly, in the study of Macko et al. 85% of patients had implant caused symptoms, 75% complained of prominent K-wires under the skin, and 20% had skin perforation^13^.

There is limited research on olecranon fracture outcomes after IM-TBW .In their multicenter review, Edwards et al.^29^ found that approximately 6% of orthopedic surgeons used IM-TBW .Murphy et al.^30^ compared commonly used methods for treatment of transverse and oblique olecranon fractures.This study included ten patients treated using IM-TBW, ten patients treated using AO tension band and 13 patients using intramedullary screw alone.The clinical evaluation showed that the IM-TBW treatment group had the best therapeutic effect. In a study on 25 patients with olecranon fractures treated with IM-TBW, 15 had excellent results (60%), 3 had good results (12%), and 7 patients had fair results (28%),and there were no poor outcomes^26^. Therefore, IM-TBW is a simple and effective method for treating olecranon fractures^26^.

Recently, we developed a cannulated screw with holes in the tail, named the Ding’s screw. In this study, we performed a biomechanical test to evaluate DSTBW and traditional TBW in the setting of olecranon fractures. Our study found that DSTBW group showed significant differences in 60°and 90° flexion angles when compared with the IM-TBW and TBW groups. In addition,the maximum failure load in the DSTBW group was significantly higher than in the IM-TBW and TBW groups. The biomechanical study on pullout force confirmed that DSTBW exhibited a notable enhancement in displacement resistance compared to IM-TBW and TBW. These results suggest that DSTBW offers greater stability and reduces the risk of internal fixation failure in olecranon fractures compared to IM-TBW and TBW models. In addition, IM-TBW offers better structural stability compared to TBW in our study, but it still has a problem with withdrawal fixation and complication of the large washer in clinical practice.The initial design of the Ding’s screw featured a diameter of 4.0 mm, which will be modified to accommodate a diameter of 6.5 mm in the future in our plan. This modification allows for the direct replacement of the conventional intramedullary screw without the need for a washer. This screw can be connected to steel wire through the holes to form tension bands, effectively reducing withdrawal fixation and soft tissue irritation symptoms associated with the use of washers in traditional IM-TBW. However, further biomechanical experiments are needed to confirm this theory.

This study has some limitations. First, the small sample size of fracture models and single testing session may impact the findings. Second, the elbow is a complex joint with synovial fluid, multiple muscles and ligaments. But the biomechanical effects of soft tissues and other bony structures such as humerus and radius were not included in our study. To study the biomechanics of elbow trauma, it is often a challenge to establish a model which is practically and ethically acceptable and also provides reliable results. Many studies have tested the biomechanical function of fixation by using fresh-frozen human cadaveric elbows^8,31,32^. However, ethical considerations to allow such cadaveric research are very difficult in our institute.Synbone models are often used in biomechanical experiments^33,34^. Their advantages are their uniform geometry and material properties, which eliminate a sample’s variability because of age, sex, anatomy, demographics, bone quality,etc.In addition, Synbone models are easier to obtain than cadaveric models.

## Conclusion

In conclusion, our biomechanical testing showed that DSTBW fixation provides more stability than TBW fixation model for olecranon fractures.

## Data Availability

All data generated or analysed during this study are included in this published article.

## Abbreviations

K-wires: Kirschner wires
TBW: Tension band wiring
DSTBW: Ding’s screw and tension band wiring
